# The Descriptive Epidemiology of Panic Attacks and Panic Disorder in Australia 2020–22: Prevalence, Correlates, Age of Onset and Changes Over Time

**DOI:** 10.1155/da/9977134

**Published:** 2026-09-28

**Authors:** Matthew Sunderland, Sam Jones, Amelia Henry, Cath Chapman, Andrew Baillie, Tim Slade

**Affiliations:** ^1^ The Matilda Centre for Research in Mental Health and Substance Use, The University of Sydney, Sydney, Australia, sydney.edu.au; ^2^ Sydney School of Health Sciences, The University of Sydney, Sydney, Australia, sydney.edu.au

**Keywords:** comorbidity, correlates, epidemiology, impairment, panic attacks, panic disorder

## Abstract

Panic disorder is highly debilitating and contributes a significant amount to anxiety‐related disability‐adjusted life years globally. To understand this burden, the current study provides an up‐to‐date understanding of the descriptive epidemiology of panic attacks and panic disorder in the Australian population. Data were from the most recent 2020–2022 National Survey of Mental Health and Wellbeing, a large‐scale population‐based study conducted in Australian households with respondents aged 16–85 years of age (*n* = 15,893). Panic attacks and panic disorder experienced across the lifetime, within the past 12 months and within the past 30‐days were assessed by the Composite International Diagnostic Interview according to the Diagnostic and Statistical Manual of Mental Disorders (DSM‐IV) diagnostic criteria to facilitate direct comparisons with the 2007 National Survey of Mental Health and Wellbeing (*n* = 8841). The lifetime prevalence of panic attacks and panic disorder was ~27% and 4%, respectively. Those with panic disorder indicate a median age of onset in their early 20s and the experience of panic disorder is not trivial, with nearly half of all cases (45%) falling within the ‘severe’ range in terms of severity and functional impairment. There were significantly elevated odds in select subgroups of the population with evidence that panic attacks and panic disorder remain a pressing concern for the Australian population, and particularly so in 2020–22 for younger cohorts (16–24), females, those who have never been married, and people who reside in inner regional areas. Despite improvements in access to mental health services, there remains a strong need for improved mental health services and targeted prevention programmes.

## 1. Introduction

Panic attacks, as outlined in the Diagnostic and Statistical Manual of Mental Disorder 5th edition (DSM‐5), are characterised by a sudden episode of intense fear or discomfort that reaches its peak within minutes and are accompanied by at least four symptoms representing physical (sweating, trembling, chest pain, nausea, etc.) and/or cognitive symptoms (derealisation, fear of dying and fear of losing control). With respect to panic disorder, a person must experience recurrent and unexpected panic attacks with at least 1 month of persistent concern/worry about having a panic attack or a change in behaviours/avoidance of places, activities or situations for fear of inducing a panic attack [1].

Panic disorder can be debilitating in terms of its physical, psychological, social and occupational impact [2, 3]. Cross‐nationally, the average prevalence of panic attacks ranges from 6.9% in low‐income countries through to 16.6% in high‐income countries, whereas the average prevalence of panic disorder was 0.8% in low‐income countries through to 2.2% in high‐income countries [4]. The highest lifetime prevalence estimates were observed in countries within the region of the Americas and the Western Pacific region. Panic disorder was found to be related to high levels of comorbidity, with 80.4% of persons who experience panic disorder globally also experiencing another disorder, and only a minority (15.4%) of whom experience panic disorder prior to the onset of another disorder [4]. High levels of substance use disorder have also been observed among people with panic disorder; for example those with panic disorder have 3.27 times the odds of having any substance use disorder and the largest association with alcohol dependence [5]. This specific comorbidity is observed as bidirectional with approx. half experiencing panic disorder prior to substance use disorder and half experienced substance use disorder prior to panic disorder, suggesting multiple explanatory and mechanistic pathways depending on specific substances used [6, 7].

In the Australian context, the 2020–2022 National Survey of Mental Health and Wellbeing recently estimated the prevalence of panic disorder at 2.7% in the past 12 months with a higher prevalence for females (3.7%) and in the younger age group (16–25; 6.0%) [8]. Co‐occurrence with other disorders was moderate to high with a comorbidity‐to‐diagnosis inflation ratio of ~1.5 (e.g. for every one case of panic disorder, there are 1.5 cases with co‐occurring disorders). The strongest correlation occurs between panic disorder and agoraphobia, social anxiety and generalised anxiety disorder [9]. Whilst the experience of panic attacks often has an earlier age of onset and higher lifetime risk, panic disorder has a later age of onset, relative to the other anxiety disorders, with a median age of 30 and a projected lifetime risk of 5% [10].

Beyond these headline rates, there is little descriptive epidemiology specific to panic disorder in the Australian population, and few studies have described the epidemiology of experiencing panic attacks (with or without panic disorder). Indeed, panic attacks can also be a sign of acute distress, with several causes, and have been proposed as a key risk marker for other psychiatric disorders, for example the odds of any non‐panic disorder in the context of panic attacks were 7.5 [11]. Existing epidemiological research was published ~13 years ago, and since then, there have been rapid changes in population mental health, including observed increases in mental disorders in general among more recent cohorts. There could be shifting risk and protective factors for panic attacks and panic disorder due to societal change and advances in treatments and prevention programmes that warrant further inspection [12, 13]. Detailed and up‐to‐date descriptive epidemiology on both panic attacks and panic disorder in the general population is required to alleviate the high degree of burden, provide evidence‐based policy and improve treatment planning for at‐risk population groups.

With the recent release of data from the Australian National Survey of Mental Health and Wellbeing 2020–22 coupled with prior data released in 2007, a more fine‐grain analysis of the descriptive epidemiology of panic attacks and panic disorder in Australia is warranted. The large sample facilities analysis within various subpopulations, including the ability to identify and track changing trends in the prevalence and correlates over time. For example, the prevalence may have remained at a similar level since 2007, but the pattern of demographic and clinical correlates may have changed. Policies and treatment practices that may have been applicable and were informed by prior data could require updating. As such, the two key aims of the current paper are to (1) provide up‐to‐date data on the descriptive epidemiology of panic attacks and panic disorder in the 2020–22 Australian National Survey of Mental Health and Wellbeing and (2) examine changes in prevalence since 2007, including investigation of the potentially moderating factors associated with change. The current analyses were not pre‐registered and should be considered exploratory in nature.

## 2. Methods

### 2.1. Sample

Data for the current study were from the 2020–22 National Survey of Mental Health and Wellbeing, an Australian Bureau of Statistics (ABS)‐administered household (private dwellings in urban and rural areas) survey of the Australian population aged between 16 and 85. Very remote parts of Australia and discrete Aboriginal and Torres Strait Islander communities were not included. The survey combined data from two cohorts collected over an 8‐month period from December 2020 to July 2021 and over an 11‐month period from December 2021 to October 2022. There were 15,893 fully responding households, representing a response rate of 52% [8]. For comparative purposes and examining the change in the intervening 13 years, the 2007 National Survey of Mental Health and Wellbeing was used. The 2007 survey utilised the same sampling framework and instrumentation to measure mental and substance use disorders and comprised 8841 households (a 60% response rate) [14]. The use of the data was approved by the ABS in line with their comprehensive data use and safety rules. Given that this is a secondary analysis of publicly available data, the current study was considered exempt from human ethics review.

### 2.2. Measurement

#### 2.2.1. Panic Attacks and Panic Disorder

Diagnostic information for panic attacks and panic disorder was obtained using the World Mental Health Consortium version of the Composite International Diagnostic Interview (WMH‐CIDI) [15]. This is a lay‐interviewer‐administered interview that has been clinically calibrated against a clinician‐administered semi‐structured interview. The lifetime and past 12‐month experience of panic attacks and panic disorder was assessed based on the criteria outlined in the fourth edition of the Diagnostic and Statistical Manual of Mental Disorders (DSM‐IV). The choice to use the DSM‐IV criteria when assessing panic disorder was made by the survey developers instead of using the more recent DSM‐5 criteria to maintain measurement consistency between the 2007 and 2020–22 surveys. However, the essential features of panic attacks remained unchanged across the two manuals except for simplification and clarification of the type of panic attacks experienced (expected vs. unexpected). For panic disorder, the major change between the manuals included the unlinking of panic disorder and agoraphobia, so as a focus of the current study, the diagnostic label of panic disorder (with or without agoraphobia) was primarily utilised to match the DSM‐5 conceptualisation of panic disorder as a single diagnostic entity. Additional disorders included major depression, dysthymia, social phobia, agoraphobia, generalised anxiety disorder, obsessive compulsive disorder, post‐traumatic stress disorder, alcohol abuse/dependence and drug abuse/dependence.

#### 2.2.2. Correlates and Service Use

Demographic and additional clinical information were obtained as part of the broader survey and used in the current study to examine correlates of panic attacks and panic disorder. These variables included age, sex at birth, country of birth, marital status, employment status, education, household composition, remoteness area (Accessibility/Remoteness Index of Australia [ARIA] defined as a measure of relative geographic access to services categorised into five classes: metro, inner regional, outer regional, remote and very remote), socioeconomic status (SEIFA; Index of relative socioeconomic disadvantage quintiles with 1 being most disadvantaged and 5 least disadvantaged), psychological distress, consultation with any health professional for a mental health problem and suicidality (suicide thoughts and behaviours). A severity measure was included to examine severity/functional impairment among those who received a diagnosis of panic disorder. A person is considered to have ‘severe’ panic disorder if they have co‐occurring bipolar disorder, experienced a suicide attempt, or experienced at least two areas of severe impairment on the Sheehan Disability Scale [16]. A person is considered to have ‘moderate’ panic disorder if they also indicate moderate (but not severe) impairment in any area on the Sheehan Disability Scale and ‘mild’ panic disorder if they indicate neither moderate nor severe impairment. The Sheehan Disability Scale is a brief 5‐item (rated on a 10‐point scale: not at all–extremely impaired) self‐report measure for impairment across work/school, social and family life due to mental health or physical symptoms.

### 2.3. Statistical Analysis

Descriptive statistics of weighted data were used to generate age‐ and sex‐specific prevalence of panic attacks and panic disorder across the lifetime, in the past 12 months, and in the past 30 days. Among those with a diagnosis, weighted estimates of the severity experienced were determined based on the severity index. The median age of onset, inter‐quartile range and projected lifetime risk of panic disorder and panic attacks were determined using the survival function based on a weighted Kaplan–Meier estimator. Significant correlates were determined by separate binary logistic regressions for panic attacks and panic disorder as the outcome variables, and age, sex, marital status, employment, country of birth, education, residence and household composition entered as predictor variables. The weighted prevalence of chronic physical conditions was calculated for those with and without panic attacks/panic disorder, and comparisons were made using odds ratios and Wilson’s 95% confidence intervals. Similarly, the weighted prevalence of health professional consultation in the past 12 months was determined for those with and without panic disorder, and statistical comparisons were made using odds ratios and Wilson’s 95% confidence intervals.

The presence of co‐occurring conditions was determined by estimating the prevalence of each additional mental health and substance use disorder among those with panic disorder. Estimated odds ratios were derived using binary logistic regression to determine the increased odds of an additional condition depending on the presence vs. absence of a diagnosis of panic attacks/panic disorder. Co‐occurrence was defined in several ways and analysed in separate models, including (1) treating all other conditions as separate and independent; (2) grouping conditions into any mood, any anxiety and any substance use disorder; (3) grouping conditions based on the number of additional disorders present (1 other, 2 others and 3 or more others); and (4) based on a single variable that determines whether any other disorder was present or not. Finally, comparisons in the prevalence of past 12‐month panic disorder across the 2007 and 2020–22 surveys and the moderating effects of sociodemographic factors were determined using binary logistic regression on a dataset that stacked both surveys together. Interactions between survey year (coded as 0 = 2007 and 1 = 2020–22 survey) and correlates of interest were estimated, and the prevalence was reported separately across the levels of those correlates that exhibited significant interactions. All weighted calculations were made using replicate weights provided by the ABS and were computed using the ‘survey’ R package [17].

## 3. Results

Lifetime, past 12‐month and past 30‐day prevalence of panic attacks and panic disorder are provided in Table 1. Approximately 27% of Australians experience panic attacks at some point in their lives, and 4.5% go on to meet criteria for panic disorder. In terms of age of onset, the median age for first meeting diagnostic criteria for panic disorder was 22 (IQR 14–42), and a projected lifetime risk of 5.8%. The prevalence of both panic attacks and panic disorder was higher for people aged 16–24 and those who were female at birth. The highest lifetime prevalence for panic attacks and panic disorder was 47% and 11% among females aged between 16 and 24 years, respectively. Additional inspection of Tables 2 and 3 indicates that females demonstrate a little over twice the odds of panic attacks and panic disorder in comparison to males. Other statistically significant correlates of panic attacks included marital status, employment, country of birth and household composition. Marital status, employment and country of birth were also significant correlates of panic disorder. To contextualise the precision of our estimates, the minimum detectable odds ratio was calculated given the achieved sample size (*n* = 15,893) and the observed prevalence rates of past 12‐month panic disorder (2.7%). As such, our study had 80% power to detect odds ratios of 1.15 or greater (or equivalently, 0.87 or smaller) at *a* = 0.05 (two‐tailed). All significant odds ratios identified in the study exceeded this threshold.

**Table 1:** Life, past 12‐month and past 30‐day prevalences of panic attacks and panic disorder across age and sex at birth in 2020–22 NSMHWB.

Age	Lifetime panic attacks					
	Males (%)	SE (%)	Females (%)	SE (%)	All (%)	SE (%)
16–24	27.25	1.71	47.38	1.87	37.04	1.34
25–44	23.23	1.10	37.99	1.34	30.70	0.88
45–64	20.14	1.21	30.01	1.30	25.17	0.84
65–85	14.04	1.17	19.36	0.91	16.82	0.68
Total	21.13	0.59	33.05	0.66	27.18	0.41
	Past 12‐month panic attacks					

16–24	14.65	1.49	30.69	1.67	22.44	1.16
25–44	8.52	0.66	15.35	0.74	11.98	0.53
45–64	5.59	0.72	10.47	0.94	8.08	0.55
65–85	2.71	0.48	5.26	0.60	4.04	0.36
Total	7.40	0.35	13.84	0.48	10.67	0.30
	Past 30‐day panic attacks					

16–24	4.66	0.87	8.22	1.04	6.39	0.66
25–44	2.36	0.39	4.31	0.47	3.35	0.36
45–64	1.43	0.36	3.22	0.54	2.35	0.35
65–85	1.35	0.36	1.99	0.34	1.68	0.22
Total	2.21	0.23	4.03	0.27	3.13	0.19
	Lifetime panic disorder					

16–24	3.62	0.65	11.15	1.23	7.28	0.73
25–44	3.65	0.45	6.75	0.62	5.22	0.41
45–64	2.65	0.47	4.41	0.59	3.55	0.39
65–85	1.51	0.33	3.13	0.48	2.36	0.30
Total	2.93	0.23	5.88	0.36	4.43	0.22
	Past 12‐month panic disorder					

16–24	3.03	0.61	9.02	1.14	5.94	0.70
25–44	2.07	0.37	4.26	0.44	3.18	0.30
45–64	1.42	0.37	2.24	0.41	1.84	0.28
65–85	0.58	0.23	1.39	0.35	1.00	0.21
Total	1.73	0.21	3.69	0.30	2.72	0.19
	Past 30‐day panic disorder					

16–24	1.49	0.46	3.61	0.65	2.52	0.39
25–44	0.62	0.18	1.65	0.30	1.14	0.19
45–64	0.78	0.30	0.92	0.26	0.85	0.21
65–85	NA	NA	NA	NA	NA	NA
Total	0.74	0.14	1.45	0.16	1.10	0.11

*Note:* Estimates with SE approaching of exceeding 50% of the point estimate (particularly for the 30‐day prevalence in older age groups) are based on small cell sizes and should be interpreted with caution. Values suppressed as ‘NA’ did not meet ABS minimum cell size requirements for data safety. = weighted percentage.

Abbreviation: SE, standard error.

**Table 2:** Correlates of past 12‐month panic attacks.

Age	Multivariate			
	OR	Lower 95% CI	Upper 95% CI	*p* (>|*t*|)
16–24	1.00	1.00	1.00	Ref
25–44	0.67	0.55	0.82	<0.001
45–64	0.42	0.33	0.55	<0.001
65–85	0.17	0.12	0.24	<0.001
*X* ^2^ (*p*‐value)	199.8	<0.001	—	—
Sex at birth				
Male	1.00	1.00	1.00	Ref
Female	2.08	1.80	2.39	<0.001
*X* ^2^ (*p*‐value)	182.5	<0.001	—	—
Marital status				
Married/de facto	1.00	1.00	1.00	Ref
Separated/divorced/widowed	1.70	1.33	2.17	<0.001
Never married	1.76	1.48	2.10	<0.001
*X* ^2^ (*p*‐value)	77.78	<0.001	—	—
Employment				
Employed	1.00	1.00	1.00	Ref
Unemployed	1.10	0.74	1.62	0.633
Not in the labour force	1.26	1.08	1.48	0.005
*X* ^2^ (*p*‐value)	11.1	0.004	—	—
Birth country				
Australia	1.00	1.00	1.00	Ref
English speaking country	0.73	0.55	0.96	0.020
Non‐English speaking country	0.49	0.40	0.60	<0.001
*X* ^2^ (*p*‐value)	97.4	<0.001	—	—
Education				
Finished high school	1.01	0.83	1.22	0.917
Did not finish high school	1.00	1.00	1.00	Ref
*X* ^2^ (*p*‐value)	0.027	0.869	—	—
Residence				
Metro	1.00	1.00	1.00	Ref
Inner regional	0.95	0.78	1.15	0.592
Outer regional	0.88	0.68	1.14	0.310
*X* ^2^ (*p*‐value)	1.90	0.387	—	—
Household composition				
Living with others	1.00	1.00	1.00	Ref
Living alone	0.86	0.73	1.02	0.082
*X* ^2^ (*p*‐value)	5.95	0.015	—	—

**Table 3:** Correlates of past 12‐month panic disorder.

Age	Multivariate			
	OR	Lower 95% CI	Upper 95% CI	*p* (>|*t*|)
16–24	1.00	1.00	1.00	Ref
25–44	0.73	0.51	1.04	0.080
45–64	0.39	0.25	0.61	<0.001
65–85	0.16	0.08	0.31	<0.001
*X* ^2^ (*p*‐value)	67.2	<0.001	—	—
Sex at birth				
Male	1.00	1.00	1.00	Ref
Female	2.15	1.58	2.94	<0.001
*X* ^2^ (*p*‐value)	53.72	<0.001	—	—
Marital status				
Married/de facto	1.00	1.00	1.00	Ref
Separated/divorced/widowed	1.83	1.13	2.97	0.015
Never married	1.76	1.25	2.48	0.001
*X* ^2^ (*p*‐value)	23.2	<0.001	—	—
Employment				
Employed	1.00	1.00	1.00	Ref
Unemployed	1.88	1.02	3.45	0.044
Not in the labour force	1.66	1.18	2.35	0.004
*X* ^2^ (*p*‐value)	21.4	<0.001	—	—
Birth country				
Australia	1.00	1.00	1.00	Ref
English speaking country	0.82	0.50	1.32	0.390
Non‐English speaking country	0.37	0.24	0.56	<0.001
*X* ^2^ (*p*‐value)	44.17	<0.001	—	—
Education				
Finished high school	1.20	0.86	1.67	0.280
Did not finish high school	1.00	1.00	1.00	Ref
*X* ^2^ (*p*‐value)	2.57	0.11	—	—
Residence				
Metro	1.00	1.00	1.00	Ref
Inner regional	0.92	0.66	1.29	0.940
Outer regional	0.49	0.63	1.78	0.850
*X* ^2^ (*p*‐value)	0.50	0.78	—	—
Household composition				
Living with others	1.00	1.00	1.00	Ref
Living alone	0.90	0.68	1.20	0.470
*X* ^2^ (*p*‐value)	0.86	0.35	—	—

With respect to co‐occurring physical conditions, those with past 12‐month panic disorder were at significantly higher odds of reporting arthritis, asthma, cancer and bronchitis or emphysema, controlling for age and sex at birth (see Table 4). Likewise, those with past 12‐month panic attacks were at significantly higher odds of receiving a diagnosis for all other specific mental disorders (except dysthymia), mental disorder groups and the number of additional co‐occurring disorders. For past 12‐month panic disorder, there were significantly higher odds of co‐occurring anxiety and affective disorders, but there was no evidence for a significant difference for co‐occurring substance use disorders (see Table 5). Approximately 80% of people with past 12‐month panic disorder also had at least one other mental or substance use disorder. Nearly half (45.4% [SE = 3.4%]) of all people with panic disorder in the past 12 months indicated their severity was ‘severe’, and 42.3% (SE = 3.4%) and 11.4% (SE = 1.8%) indicated ‘moderate’ and ‘mild’ severity, respectively. It should be noted that precision varies across subgroups, with wider confidence intervals observed for older age groups and less common outcomes (e.g. 30‐day prevalence). Caution is warranted when interpreting findings for 65–85‐year‐olds with recent panic disorder, where small cell sizes resulted in some estimates being suppressed for data safety reasons.

**Table 4:** Chronic physical conditions and past 12‐month panic disorder.

Condition	%	SE	OR	Lower 95% CI	Upper 95% CI
Arthritis	17.13	2.27	2.03	1.35	3.06
Osteoporosis	2.54	0.75	0.78	0.38	1.63
Asthma	20.39	1.78	1.78	1.41	2.25
Cancer	4.86	1.20	1.87	1.02	3.44
Dementia	NA	NA	NA	NA	NA
Diabetes	6.57	2.01	1.68	0.82	3.44
Heart disease	3.99	0.98	1.01	0.58	1.76
Stroke	NA	NA	NA	NA	NA
Chronic kidney	NA	NA	NA	NA	NA
Bronchitis or emphysema	5.42	1.39	2.96	1.62	5.44
Any‐long term condition	79.64	2.14	5.38	4.07	7.10

*Note:* Odds ratios generated in logistic regression models controlling for age and sex at birth. NA = values were suppressed due to ABS data safety rules.

**Table 5:** Models of co‐occurrence with past 12‐month panic attacks and panic disorder.

Panic attack (with or without panic disorder)						
Co‐morbid condition	*N*	%	S.E.	OR	95% CI	95% CI
Major depression	481	31.88	1.44	4.01	2.99	5.37
Dysthymia	158	10.51	1.01	0.84	0.49	1.41
Social phobia	504	33.59	1.65	3.97	3.16	4.99
Agoraphobia	193	13.30	1.16	2.79	1.70	4.58
GAD	359	22.69	1.16	2.62	1.99	3.44
OCD	208	14.18	1.04	2.13	1.50	3.03
PTSD	249	14.15	0.99	2.04	1.46	2.84
Alcohol abuse or dependence	111	7.23	0.86	1.98	1.34	2.92
Drug abuse or dependence	18	1.09	0.28	2.06	1.06	4.01
Disorder groups						

Any affective	973	33.00	1.43	3.53	2.78	4.49
Any anxiety	499	54.42	1.87	6.43	5.32	7.77
Any substance use	145	9.64	0.94	2.11	1.61	2.76
Number of disorders						

1 other disorder	388	25.07	1.38	6.24	5.17	7.52
2 other disorders	242	14.77	1.07	13.57	10.77	17.09
> = 3 other disorders	369	24.41	1.45	28.40	22.82	35.34

Any other disorder	312	64.25	1.46	10.77	9.28	12.49

**Panic disorder**						

**Co-morbid condition**	***N***	**%**	**S.E.**	**OR**	**95% CI**	**95% CI**

Major depression	163	43.19	2.77	2.68	1.69	4.26
Dysthymia	64	17.43	2.31	1.15	0.66	2.01
Social phobia	184	49.93	3.39	4.36	2.96	6.42
Agoraphobia	90	24.48	2.67	2.79	1.77	4.39
GAD	133	35.29	2.54	2.64	1.79	3.90
OCD	70	19.14	2.31	1.70	1.05	2.74
PTSD	81	18.96	2.32	1.49	0.81	2.71
Alcohol abuse or dependence	35	9.60	2.11	1.69	0.88	3.23
Drug abuse or dependence	NA	NA	NA	NA	NA	NA
Disorder groups						

Any affective	169	44.20	2.75	1.91	1.51	2.43
Any anxiety (other than PDS)	281	73.35	2.82	10.86	7.73	15.30
Any substance use	46	12.73	2.25	1.55	1.00	2.41
Number of disorders						

1 other disorder	91	22.22	2.68	8.49	5.94	12.13
2 other disorders	73	18.47	1.97	22.11	15.22	32.12
> = 3 other disorders	148	39.74	3.24	48.16	35.65	65.05
Any other disorder	312	80.43	2.12	18.80	14.31	24.69

Consultations with any mental health and health services were significantly higher among those with past 12‐month panic disorder, with an approximate 9‐fold increase (OR = 8.7, 95% CI = 6.7–11.4) in the odds of any service use and a 12‐fold increase (OR = 11.9, 95% CI = 6.3–22.4) in hospital admissions for any mental health‐related condition in comparison to those without panic disorder. As can be seen in Table 6, the odds of consultations with specific mental health professionals were significantly increased among those with panic disorder in the past 12 months compared to those without.

**Table 6:** Service utilisation among those with a diagnosis of past 12‐month panic disorder.

Service use	%	SE	OR	95% CI	95% CI
General practitioner	54.98	3.22	8.00	6.05	10.57
Psychiatrist	15.99	2.18	5.42	3.70	7.95
Psychologist	30.81	2.45	4.47	3.39	5.89
Mental health nurse	4.94	1.19	9.16	4.71	17.82
Specialist doctor or surgeon	2.36	0.83	5.49	2.52	11.97
Other mental health professional	19.52	3.24	6.48	3.99	10.54
Other health professional	3.68	1.33	4.43	1.89	10.38
Any consultation with a mental health professional	48.36	2.64	6.18	4.85	7.87
Any consultation with a health professional	66.39	2.86	8.76	6.68	11.48
Admission for any mental health condition	5.35	1.46	11.87	6.29	22.39
Any service use	66.39	2.86	8.70	6.65	11.39

The binary logistic regressions examining differences across time (survey year) generated evidence for a significant interaction associated with age (*F* = 5.42, *p* = 0.002), sex at birth (*F* = 4.71, *p* = 0.032), marital status (*F* = 4.71, *p* = 0.011), and remoteness area (*F* = 5.79, *p* = 0.004). The predicted probability of past 12‐month panic disorder and 95% confidence intervals estimated separately for the levels of age, sex at birth, marital status and remoteness area are provided in Figures 1–4, respectively. Overall, the past 12‐month prevalence of panic disorder has shifted from 1.8% (SE = 0.2) in 2007 to 2.7% (SE = 0.2) in 2020–22. Moreover, the type of person who experiences panic disorder in the past 12 months in 2020–22 compared to 2007 has significantly changed, with increased odds of panic disorder in 2020–22 found for those who are younger (16–24), female, never married and from inner regional areas.

**Figure 1:**
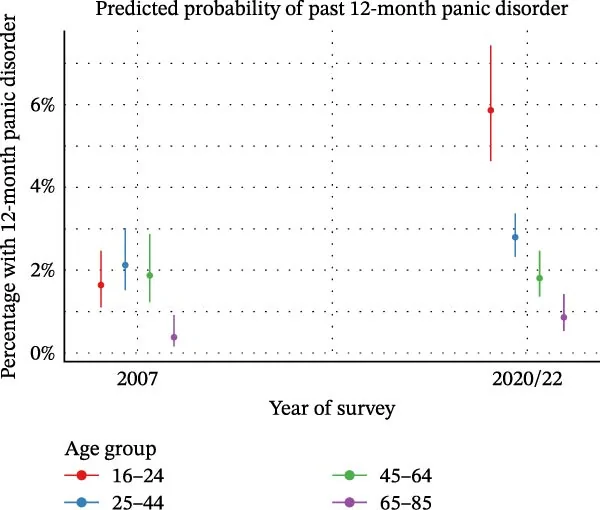
Predicted probability of past 12‐month panic disorder by year of survey and age group.

**Figure 2:**
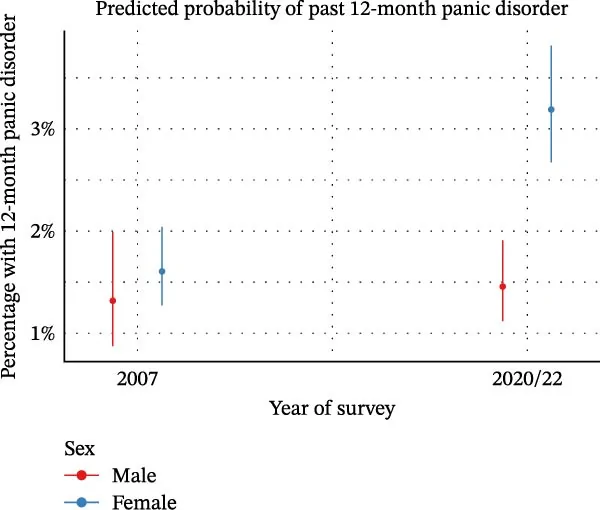
Predicted probability of past 12‐month panic disorder by year of survey and sex at birth.

**Figure 3:**
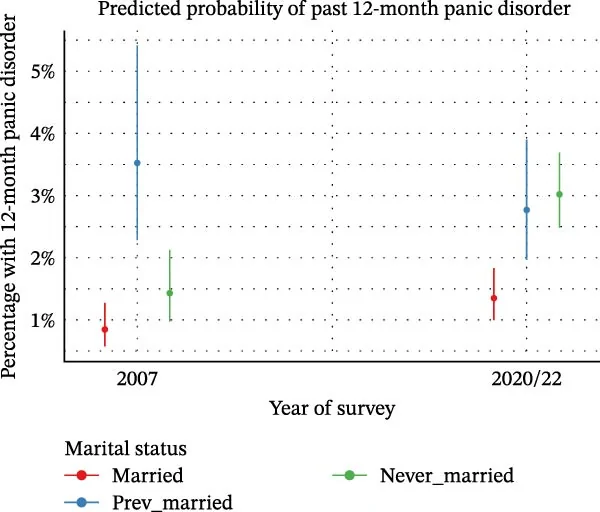
Predicted probability of past 12‐month panic disorder by year of survey and marital status (married, previously married and never married).

**Figure 4:**
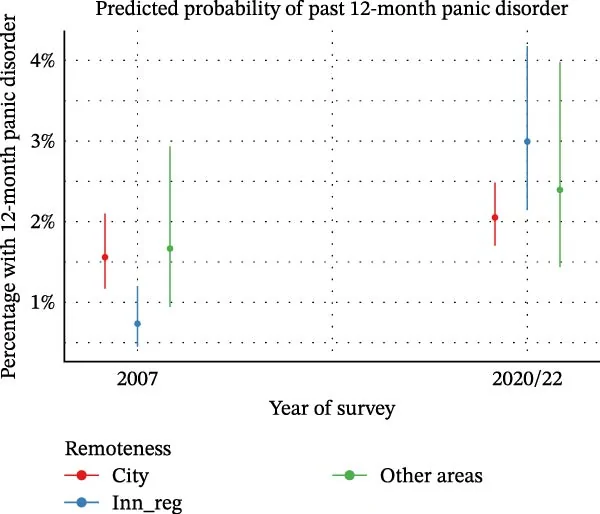
Predicted probability of past 12‐month panic disorder by year of survey and remoteness (city, inner regional and other areas).

## 4. Discussion

The current study aimed to provide a contemporary and comprehensive picture of the descriptive epidemiology of DSM‐IV panic attacks and panic disorder in the Australian population and draw comparisons with the estimates determined in 2007. Overall, the lifetime prevalence of panic attacks and panic disorder in the population remains consistently high, with ~27% and 4%, respectively. Those with panic disorder indicate a median age of onset in their early 20s, which worryingly appears to be decreasing in comparison to the median age of onset of 30, last estimated in 2007 [10]. The current prevalence continues to place the Australian population in the upper ranks for both panic attacks and panic disorder experienced across all high‐income countries assessed globally [4]. Moreover, the experience of panic disorder is not trivial, with nearly half of all cases (45%) falling within the ‘severe’ range in terms of severity and functional impairment. Higher odds of panic disorder were found among people who are younger (16–44), female, not currently married or in a de facto relationship, not currently employed, or those born in Australia. Similarly, the odds of any co‐occurring mental health and/or any co‐occurring chronic physical conditions were significantly elevated in those with past 12‐month panic disorder in comparison to those who do not have panic disorder. This finding is also reflected in significantly elevated odds in treatment seeking for service use and mental health hospital admissions; however, there is some uncertainty regarding how much these elevated odds are directly related to panic disorder vs other comorbid conditions. Combined, these results provide evidence that panic attacks and panic disorder remain a pressing concern for the Australian population, and particularly so in 2020–22 for younger cohorts (16–24), females, those who have never been married, and people who reside in inner regional areas. An important consideration is that this study used DSM‐IV criteria, which conceptualised panic disorder as a single diagnostic entity ‘with or without agoraphobia’, whereas DSM‐5 dropped this distinction. In the current study, the DSM‐IV diagnosis of panic disorder was determined regardless of the presence of agoraphobia and therefore provides a close comparison with the recent DSM‐5 criteria. However, additional minor wording changes were implemented in the criteria for both panic attacks and panic disorder. As such, direct comparisons with DSM‐5‐based studies require caution. These diagnostic differences should be considered when interpreting trends over time and planning services based on our prevalence estimates.

The findings presented in the current study reflect a broader trend observed in the recent literature regarding increases in the prevalence of mental disorders, particularly mood and anxiety disorders [18]. These results also suggest that the increases in panic disorder observed from 2007 are particularly relevant and differentially impacting those aged 16–24, female, those who have never been married, and those who live in an inner regional area. The notable decrease in age of onset appears to reflect this trend, with more recently born cohorts (who are younger at time of survey) indicating an earlier age of onset from those surveyed in 2007. There could be potential differences in risk and protective factors that have emerged since 2007 that differentially impact these groups of the population. Some studies have reported increases in loneliness and reduced social networks [19], changes in parenting style and intergenerational transmission of poor mental health [20], increased access to and use of social media [21], and changes in broad lifestyle behaviours (increased sedentary behaviour and reduced physical activity and sleep) [22–24]. These changes may be potential mechanisms that have led to specific increases in poor mental health and the observed increases in panic attacks and panic disorder found in the current study. However, further research is required, specifically longitudinal studies that directly assess the causal relationships across relevant factors and outcomes, including changes in mental health literacy, reducing stigma, government initiatives to improve access to mental health services, and increased understanding of the risk and protective factors, including neurochemical, genetic and epigenetic factors [25].

The timing of the most recent survey during 2020 and 2022 aligns with the global COVID‐19 pandemic that resulted in substantial disturbance to daily life, increased financial hardships due to job losses, and significant burden associated with new lockdown laws and social distancing that may have contributed to elevated past 12‐month and past 30‐day prevalence estimates. Indeed, additional estimates have demonstrated that the COVID‐19 pandemic was associated with significant increases in uncertainty, life disruption, loss of income, anxiety, and psychological distress [26, 27]. The key question remains as to whether the increases observed in panic attack and panic disorder prevalence in the current study were overly influenced by the pandemic in comparison to rates that may have been generated if the pandemic did not occur. This is a difficult counter‐factual question to answer and one that required additional research; however, two large systematic reviews have concluded that the pandemic may have contributed only small to moderate increases in the prevalence of anxiety and depression over the short term [28, 29]. Likewise, longitudinal evidence suggests that rates of psychological distress have been trending upwards for several years prior to the pandemic, and these studies have suggested that broader contextual and social changes are more likely the drivers of increased prevalence of anxiety [30–32].

There are several strengths to the current study, including the use of two large‐scale population‐based surveys, the use of the same sampling and consistent assessment methods across both surveys to facilitate comparisons, and the comprehensive evaluation of both DSM‐IV panic attacks and panic disorders as separate diagnostic entities. However, there are several limitations that require additional discussion. The two surveys had lower than optimal response rates of 60% and 52%, respectively. This could introduce a potential non‐response bias that is only partially mitigated by calibration weighting. If persons with panic attacks or panic disorder were less likely to participate, the prevalence estimates and associations may be conservative. The ABS has not formally published a non‐response bias analysis for the 2020–2022 wave, but a comparable analysis is available from the 2007 survey and identified possible underestimation among men and young people. The results also represent cross‐sectional and retrospective reporting of symptoms experienced across the lifespan, which could be influenced to some degree by the recall bias. The results should also not be interpreted as causal, given the cross‐sectional nature of the data.

While our large overall sample size (*n* = 15,893) provides robust estimates for the total population, precision is limited for some subgroup analyses, particularly for older age groups with recent (30‐day) outcomes. Replication in targeted studies of older adults would strengthen confidence in age‐specific patterns. All associations are reported as odds ratios with 95% confidence intervals. Given the descriptive and exploratory nature of the study, *p*‐values are reported without any adjustment for multiple comparisons; confidence intervals are the primary basis for inference. Findings should be considered as hypothesis‐generating pending replication in independent samples, and findings with marginal statistical significance should be treated with caution. Studies that seek to develop theoretical frameworks of the development of panic disorder would benefit from specifically testing key aspects of the theory in relation to the empirical evidence regarding panic disorder [33]. Finally, despite extensive adjustment for sociodemographic and clinical covariates, there is the possibility that some of the associations identified in the current study are partially confounded by unmeasured lifestyle factors. For example, the observed association between unemployment and panic disorder (OR = 1.88) may be partially confounded by unmeasured lifestyle factors (e.g. disrupted sleep patterns and reduced physical activity) that are both consequences of unemployment and risk factors for panic disorder. In future work, the significant associations found in the current study need to be replicated with additional confounders controlled for in the models.

Nonetheless, the current study provides updated information regarding the prevalence, correlates, service use, comorbidity, and severity of panic attacks and panic disorder experienced by the Australian population. The results demonstrate that panic attacks and panic disorder have remained consistently high since 2007 and have specifically increased in younger cohorts (aged 16–24 years), females, those who have never been married, and those from inner regional communities. The results provide evidence of changing correlates associated with panic disorder over time that may indicate changing or emerging risk factors and protective factors that require further investigation. Finally, despite improvements in access to mental health services, there remains a strong need for improved mental health services and targeted prevention programmes for panic attacks that could potentially reduce overall psychopathology in the future.

## Funding

No funding was received for this manuscript.

## Disclosure

The work was performed as part of the employment of the authors at the University of Sydney.

## Conflicts of Interest

The authors declare no conflicts of interest.

## Data Availability

The data that support the findings of this study are available from the Australian Bureau of Statistics. Restrictions apply to the availability of these data, which were used under a licence for this study. Data are available from https://www.abs.gov.au/ with the permission of the Australian Bureau of Statistics.
